# Patterns Testing for Tick-Borne Diseases and Implications for Surveillance in the Southeastern US

**DOI:** 10.1001/jamanetworkopen.2022.12334

**Published:** 2022-05-16

**Authors:** Amanda Brown Marusiak, Brandon D. Hollingsworth, Haley Abernathy, Aidin Alejo, Victor Arahirwa, Odai Mansour, Dana Giandomenico, John Schmitz, Carl Williams, Alexis M. Barbarin, Ross M. Boyce

**Affiliations:** 1 Department of Epidemiology, Gillings School of Global Public Health, University of North Carolina at Chapel Hill, Chapel Hill, North Carolina; 2 Institute for Global Health and Infectious Diseases, University of North Carolina at Chapel Hill, Chapel Hill, North Carolina; 3 Department of Pathology and Laboratory Medicine, McLendon Clinical Laboratories, UNC Health, Chapel Hill, North Carolina; 4 Division of Public Health, Communicable Disease Branch, Raleigh, North Carolina; 5 School of Medicine, University of North Carolina at Chapel Hill, Chapel Hill, North Carolina; 6 Carolina Population Center, University of North Carolina at Chapel Hill, Chapel Hill, North Carolina

## Abstract

**Importance:**

Tick-borne diseases (TBD), including spotted fever group rickettsiosis (SFGR), ehrlichiosis, and, increasingly, Lyme disease, represent a substantial public health concern throughout much of the southeastern United States. Yet, there is uncertainty about the epidemiology of these diseases because of pitfalls in existing diagnostic test methods.

**Objective:**

To examine patterns of diagnostic testing and incidence of TBD in a large, academic health care system.

**Design, Setting, and Participants:**

This cross-sectional study included diagnostic test results for TBD at UNC Health, a large academic health care system with inpatient and outpatient facilities, from January 1, 2017, to November 30, 2020. Participants included all individuals seeking routine care at UNC Health facilities who had testing for SFGR, ehrlichiosis, or Lyme disease performed during the study period.

**Main Outcomes and Measures:**

Rates of test positivity, testing completeness, and incidence of TBD.

**Results:**

During the 4-year study period, 11 367 individuals (6633 [58.4%] female; 10 793 [95%] non-Hispanic individuals and 8850 [77.9%] White individuals; median [IQR] age, 53 [37-66] years) were tested for TBD. Among the 20 528 diagnostic tests performed, 47 laboratory-confirmed, incident cases of SFGR, 27 cases of ehrlichiosis, and 76 cases of Lyme were confirmed, representing incidence rates of 4.7%, 7.1%, and 0.7%, respectively. However, 3984 of SFGR tests (79.3%) and 3606 of *Ehrlichia* tests (74.3%) lacked a paired convalescent sample. Of 20 528 tests, there were 11 977 tests (58.3%) for Lyme disease from 10 208 individuals, 5448 tests (26.5%) for SFGR from 4520 individuals, and 3103 tests (15.1%) for ehrlichiosis from 2507 individuals. Most striking, testing for ehrlichiosis was performed in only 55% of patients in whom SFGR was ordered, suggesting that ehrlichiosis remains underrecognized. An estimated 187 incident cases of SFGR and 309 of ehrlichiosis were potentially unidentified because of incomplete testing.

**Conclusions and Relevance:**

In this cross-sectional study, most of the patients suspected of having TBD did not have testing performed in accordance with established guidelines, which substantially limits understanding of TBD epidemiology. Furthermore, the data revealed a large discrepancy between the local burden of disease and the testing performed. These findings underscore the need to pursue more robust, active surveillance strategies to estimate the burden of TBD and distribution of causative pathogens.

## Key Points

**Question:**

What is the tick-borne disease diagnostic testing landscape in central North Carolina?

**Findings:**

In this cross-sectional study of 11 367 individuals, there was a higher incidence of spotted fever group rickettsiosis (SFGR) (4.7%) and ehrlichiosis (7.1%) relative to Lyme disease (0.7%), which had more than twice the testing volume. Most of those tested for SFGR and ehrlichiosis lacked convalescent testing, and the test volume for SFGR was twice that for ehrlichiosis despite a similar clinical spectrum.

**Meaning:**

These findings suggest that ehrlichiosis remains underrecognized and Lyme disease test ordering is overrepresented given its low incidence.

## Teaser

This cross-sectional study examines patterns of diagnostic testing for and incidence of tick-borne diseases in North Carolina.

## Introduction

From 2004 to 2016, the US Centers for Disease Control and Prevention (CDC) received nearly 500 000 reports of tick-borne diseases (TBD), comprising more than three-quarters of all vector-borne disease reports in the continental United States.^1^ Notably, reports of TBD doubled during this same time period, with consistent annual increases in Lyme disease, spotted fever group rickettsiosis (SFGR), and ehrlichiosis. These estimates, based on reports from state health departments, likely capture only a fraction of total infections and clinical illness.^2,3,4^ These trends will likely be exacerbated by global climate change, which may affect not only the geographic distribution of ticks, but also the tick and pathogen life cycles.^5,6,7^

While TBD represents an emerging public health crisis, timely and accurate surveillance is severely constrained by both the passive nature of reporting^8^ and limitations of existing diagnostic methods.^9^ Indirect immunofluorescence assays (IFA) are widely employed laboratory tests for TBD which rely on detection of host antibodies to the infecting pathogen.^10^ Because antibodies are often not present at high levels during the first week of illness—the period when most patients initially seek care—a negative test cannot rule out disease. To confirm the diagnosis of SFGR or ehrlichiosis, a second, or convalescent, test is required 2 to 10 weeks after the initial, or acute, test. Unfortunately, few patients undergo both acute and convalescent testing with less than 3% of SFGR cases reported to the CDC being classified as confirmed.^11^ In the absence of paired acute and convalescent results, interpretation of a single titer is challenging, especially in areas where background rates of seropositivity are high.^12^

In contrast, testing for Lyme disease employs a 2-tier algorithm on a single serum sample, consisting of an enzyme immunoassay (EIA) or IFA, followed by a Western blot if the EIA is positive or equivocal.^13^ While this approach does not require multiple samples collected at different time points, clinical practice, especially in historically lower-incidence states, is fraught with ordering errors (ie, Western blot without a preceding EIA) and misinterpretation of results.^14^ Furthermore, incorporation of the much less specific IgM-based assay, intended to increase sensitivity early in the disease course, often increases false positivity rates.^15,16^

Diagnosis and prevention of TBD depends greatly on an accurate understanding of local TBD epidemiology, much of which is derived from routine clinical practice, thus improving clinician adherence to diagnostic testing guidelines remains a key priority.^17^ Without new testing methods or rigorous protocols to ensure high levels of testing completeness, estimates of disease incidence will remain susceptible to both under- and overrepresentation, the magnitude of which is ill-defined. Perhaps, more importantly, misunderstanding of the predictive value of test results, especially a negative acute result, may contribute to delays in diagnosis and treatment, which is one of the main risk factors for severe disease and death.^18,19,20^

To date, there has been a relative paucity of research examining the patterns of diagnostic testing for tick-borne disease, particularly for SFGR and ehrlichiosis. Moreover, previous studies have largely focused on data derived from routine surveillance, which does not include individuals who test negative. Including individuals who test negative provides important information about the underlying population assumed to be at risk and can yield complementary information regarding disease incidence and routine clinical practice. Therefore, the overarching objective of this study was to explore patterns in diagnostic testing for TBD in a large academic health system with the goals of (1) describing the demographic characteristics of patients tested for TBD, (2) determining rates of adherence to testing guidelines, and (3) identifying potential gaps in clinician ordering behaviors that may be improved with systems-based interventions (eg, panels, reflex testing).

## Methods

This cross-sectional study was approved by the institutional review board at the University of North Carolina at Chapel Hill. Written informed consent or a waiver of Health Insurance Portability and Accountability Act authorization was not required because the data were classified as a limited data set under the Code of Federal Regulations 45, Part 164.514 (e). This study follows the Strengthening the Reporting of Observational Studies in Epidemiology (STROBE) reporting guideline.

### Study Site

North Carolina experiences some of the highest rates of SFGR and ehrlichiosis in the United States, often accounting for more than of 10% and 5% of the national totals reported to the CDC, respectively.^11^ In 2019, the incidence rate of SFGR was 6.6 cases per 100 000 residents with most cases being geographically clustered in the central and eastern parts of the state.^21^ Ehrlichiosis is less frequently reported, but is an underrecognized cause of tick-borne illness, despite evidence that infection rates may be similar to those of SFGR.^18^ Cases of Lyme disease are increasing in the state and are more concentrated in northwestern parts of the state in proximity to the Appalachian Mountains.^22,23^

We obtained diagnostic test results from the Carolina Data Warehouse for Health, a central repository containing clinical, research, and administrative data sourced from the UNC Health, the largest academic health system in North Carolina, comprising 12 hospitals and 350 outpatient clinics located across the state. In 2018, UNC Health reported approximately 3.5 million clinical visits, including approximately 500 000 emergency department visits.

### Data Sources and Management

We requested the results of diagnostic testing performed by UNC’s McLendon Clinical Laboratories for SFGR, ehrlichiosis, and Lyme disease from January 1, 2017, to November 30, 2020. Specifically, we abstracted results of IFA testing for immunoglobulin G (IgG) antibodies against *R. rickettsia* and *E. chaffeensis,* recognizing that cross-reactivity between species is well-known.^24^ Immunoglobulin M (IgM) results for *Rickettsia* or *Ehrlichia,* when ordered, were excluded from the analysis given that these tests are not included in the current testing guidelines. For Lyme disease, we abstracted the results of EIA, Western blots and polymerase chain reaction (PCR). Western blot results were included in the analysis regardless of the preceding EIA result and even if the EIA was not ordered or not available.

For each result, we documented the date of the test and age, sex, race, and ethnicity of the patient. Sex, race, and ethnicity data originated from medical records. Race and ethnicity were self-identified by the patients at intake and were considered to compare the TBD-tested population with the general patient population at UNC Health. Race categories included American Indian or Alaskan Native, Asian, Black or African American, White, and other, with other race representing individuals who did not identify with a provided category. Ethnicity categories included Hispanic or Latino, not Hispanic or Latino, and unknown.

### Statistical Analysis

Data were cleaned to remove duplicate entries. All analysis was performed using R version 4.0.2 (R Project for Statistical Computing). We compared test positivity for each disease across age and sex using Wilcoxon rank sum tests and Pearson χ^2^ tests, respectively, with a significance level of *P* < .05. χ^2^ tests were performed for differences in test volume by season, defined as high (March to October) and low season (November to February) based on historical tick activity,^21^ with a null assumption of equal testing throughout the year.

We established operational case classifications and laboratory criteria (Table 1) modeled after the CDC’s 2020 Surveillance Case Definitions used for SFGR and ehrlichiosis.^25,26^ We excluded clinical criteria from these definitions due to lack of clinical information. Tests for SFGR and ehrlichiosis were considered paired if the acute and convalescent samples were performed on the same individual between 14 and 70 days of each other.^24^ Paired tests were subsequently defined to represent an incident case if either (1) the result of the acute test was less than or equal to 1:64 and the result of the convalescent test was greater than or equal to 1:64 for ehrlichiosis or greater than or equal to 1:128 for SFGR or (2) the results of both acute and convalescent test were greater than or equal to 1:64, and the convalescent result represented at least a 4-fold increase in titer compared with the acute result. Paired tests were defined as a prevalent case if the convalescent titer was less than 4-fold that of the acute and (1) both tests were greater than or equal to 1:64 for ehrlichiosis or (2) both tests were greater than or equal to 1:64 with at least 1 test greater than or equal to 1:128 for SFGR. In addition, we defined an individual as a probable case if at least 1 dilution was greater than or equal to 1:128 or a suspected case if at least 1 test was positive at 1:64. A patient was considered as having a Lyme disease case if (1) their EIA test result was indeterminate or positive and the Western blot satisfied criteria for either IgG or IgM positivity, (2) they lacked an antibody test performed and their Western blot was positive for IgG, or (3) they had a positive PCR result from cerebrospinal fluid (CSF).

**Table 1.** Operational Use of Diagnostic Terminology and Case Classifications for SFGR and Ehrlichiosis Test Results Used Throughout the Study

Term	Operational definition
Paired test	Acute and convalescent IFA testing performed within 14-70 d
Incident case	Paired tests with 4-fold increase in titer from acute to convalescent sample OR Paired tests with change from negative (<1:64) to positive (≥1:64 for erhlichiosis, ≥1:128 for SFGR) from acute to convalescent sample; also referred to as seroconversion
Prevalent case	Paired positive tests with less than 4-fold change in titer between acute and convalescent test, both tests ≥1:64 for ehrlichiosis, and at least one test ≥1:128 for SFGR
Suspected case	At least one positive test result at ≥1:64
Probable case	At least one positive test result at ≥1:128
Negative	No positive test result at ≥1:64

Abbreviations: IFA, indirect fluorescent antibody; SFGR, spotted fever group rickettsiosis.

## Results

During the study, a total of 20 528 diagnostic tests for TBD were performed on 11 367 unique individuals, including 11 977 tests for Lyme disease from 10 208 individuals, 5448 tests for SFGR from 4520 individuals, and 3103 tests for ehrlichiosis from 2507 individuals (Table 2).

**Table 2.** Demographics for Individuals Tested and Testing Positive for Tick-Borne Disease Within the UNC Health System, 2017-2020

Demographic	Participant, No. (%)						
	Overall	Lyme		SFGR		Ehrlichiosis	
		Tested	Positive	Tested	Incident	Tested	Incident
Individuals	11 367	10 208	76	4520	25	2507	27
Sex							
Male	4734 (41.6)	4150 (40.7)	36 (47.4)	2137 (47.3)	12 (48.0)	1206 (48.1)	13 (48.1)
Female	6633 (58.4)	6058 (59.3)	40 (52.6)	2383 (52.7)	13 (52.0)	1301 (51.9)	14 (51.9)
Age median (IQR)	53 (37-66)	53 (38-66)	50 (30-62)	52 (36-66)	57 (45-68)	53 (38-67)	61 (38-73)
Race^a^							
American Indian or Alaska Native	56 (0.5)	49 (0.5)	0	0	0	14 (0.6)	0
Asian	116 (1.0)	101 (9.9)	0	45 (1.0)	0 (2.1)	24 (1.0)	0
Black or African American	1282 (11.2)	1137 (11.1)	8 (10.5)	408 (9.0)	2 (8.0)	217 (8.7)	0
White	8850 (77.9)	7993 (78.3)	59 (77.6)	3656 (80.9)	22 (88.0)	2027 (80.8	25 (92.6)
Other^b^	686 (6.0)	591 (5.8)	3 (3.9)	277 (6.1)	0	161 (6.4)	1 (3.7)
Unknown	377 (3.3)	337 (3.3)	6 (7.9)	134 (3.0)	1 (4.0)	64 (2.6)	1 (3.7)
Ethnicity^a^							
Hispanic or Latino	574 (5.0)	494 (4.8)	3 (3.9)	242 (5.4)	0	140 (5.6)	1 (3.7)
Not Hispanic or Latino	10 288 (90.5)	9272 (90.8)	68 (89.5)	4096 (90.6)	24 (98.0)	2273 (90.7)	25 (92.6)
Unknown	505 (4.4)	442 (4.3)	5 (6.6)	182 (4.0)	1 (2.0)	94 (3.7)	1 (3.7)

^a^ Self-reported race and ethnicity data abstracted from medical record.

^b^ Other race includes any individual identifying with a race outside the provided categories.

The median (IQR) age of individuals tested was 53 (37-66) years. Children younger than 18 years represented 671 (5.9%) of those tested, much lower than the UNC Health systemwide under 20 patient population in 2017 (195 971 of 1 027 521 [19%]).^27^ Women represented 6633 (58.4%) of individuals tested, similar to the demographic profile of patients systemwide (587 768 of 1 011 507 [58.1%]), while 10 288 of 11 367 (90.5%) identified as non-Hispanic individuals (833 171 of 1 011 649 [82.9%] systemwide) and 8850 of 11 367 (77.9%) as White individuals (628 131 of 1 011 649 [62.1%] systemwide). Racial and ethnic breakdowns did not vary substantially between those tested for each disease.

A total of 4144 of 11 367 individuals (36.5%) were tested for more than 1 TBD with combinations Lyme, SFGR, and ehrlichiosis (1724 [15.2%]) and Lyme and SFGR (1686 [14.8%]), being most frequent. Testing for SFGR and ehrlichiosis was significantly higher during the high season (March-October) with 4785 (87.9%; *P* < .001) and 2683 (86.5%; *P* < .001) of tests occurring during this period, respectively (Figure). In contrast, testing for Lyme was more equally distributed throughout the year. When stratified by the year testing was performed (eTable 1 in the Supplement), we observed a large decline in total tests performed in 2020, even when accounting for missing December 2020 data.

**Figure.**
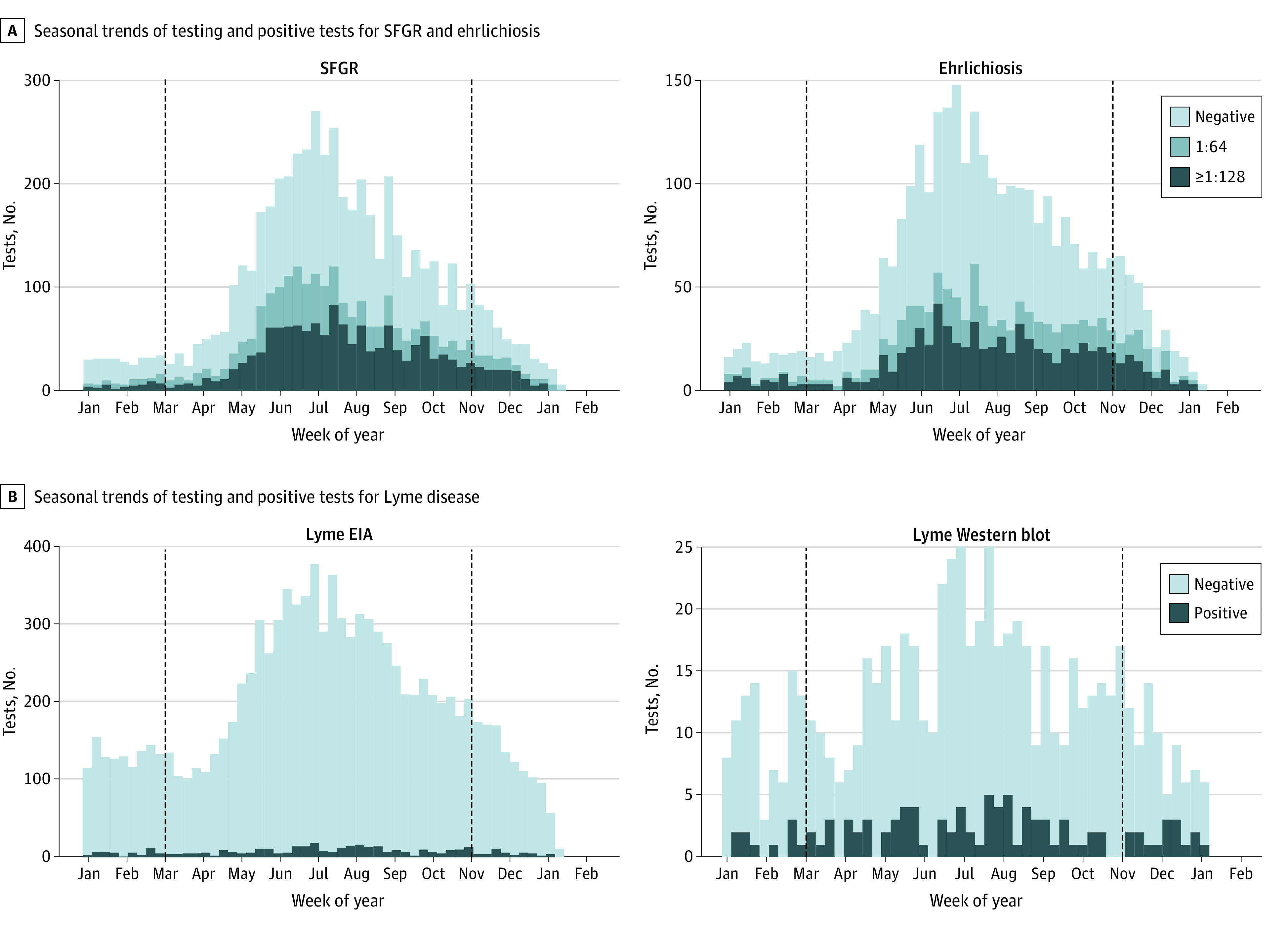
Seasonal Trends of Testing and Positive Tests for SFGR, Ehrlichiosis, and Lyme Disease in North Carolina, 2017-2020 Tests are aggregated by week of the year, with weekly number of tests given by the height of the bar and test result denoted by the color. Tick season (March-October) is designated by the vertical dashed lines. For Lyme disease, equivocal EIAs are included with positive results. EIA indicates enzyme immunoassay; SFGR, spotted fever group rickettsiosis.

The overall test positivity rate (TPR), defined as the proportion of all tests that were positive at greater than or equal to 1:128, was 27.1% for SFGR and 38.8% for ehrlichiosis, reflecting positive results of greater than or equal to 1:64. The TPR for Lyme disease, defined as the proportion of positive EIA, PCR, and Western blot results, was 4.2%. TPR increased substantially during the analysis period for SFGR and for ehrlichiosis, which more than doubled from 16.7% to 53.2%. In contrast, TPR for Lyme disease remained relatively stable. Assuming clinical criteria were met, among individuals with paired tests, there were 25 (4.7%) incident cases of SFGR and 27 (7.1%) incident cases of ehrlichiosis (Table 3). A total of 76 (0.7%) individuals satisfied the 2-tier testing criteria for Lyme disease, among which 11 met both IgM and IgG criteria, 12 met only IgG criteria, and 53 met only IgM criteria (Table 4; eTable 2 in the Supplement). None of the 224 Lyme disease PCR tests conducted on CSF were positive.

**Table 3.** Number of Tests and Paired Testing Completeness, SFGR, and Ehrlichiosis

Characteristic	Test, No. (%)	
	SFGR	Ehrlichiosis
Individuals	4520	2507
Individuals with paired tests	536 (11.8)	378 (15.1)
Incident case	25 (4.7)	27 (7.1)
Seroconversion	19 (76.0)	17 (63.0)
4-fold increase	6 (24.0)	10 (37.0)
Prevalent case	267 (49.8)	239 (63.2)
Probable case	626 (13.8)	384 (15.3)
Suspected case	288 (6.4)	233 (9.3)
Total tests	5448	3103
Positive tests	2396 (44.0)	1206 (38.8)
1:128 threshold	1475 (61.6)	290 (24.0)
No paired test	1517 (27.8)	640 (53.1)
Negative tests	3029 (55.6)	1897 (61.1)
Equivocal	23 (0.4)	0
Paired tests (total)	1128 (20.7)	797 (25.7)
Tests without a convalescent pair, No.	4320 (79.3)	2306 (74.3)

Abbreviation: SFGR, spotted fever group rickettsiosis.

**Table 4.** Serial Testing Results for Individuals Tested for Lyme Disease, Enzyme Immunoassay (EIA), and Western Blot (WB)

Test	IgG+		IgG–		WB Not performed
	IgM+	IgM-	IgM+	IgM–	
EIA +	11^a^	12^a^	53^a^	243	17
EIA –	1	0	6	95	9420
EIA not performed	0^a^	0	5	188	157^b^

Abbreviations: EIA, enzyme immunoassay; IgG, immunoglobulin G; IgM, immunoglobulin M; WB, western blot.

^a^ Colored cells denote those meeting the Lyme disease case definition in our analysis.

^b^ Individuals missing both EIA and Western blot results received only PCR testing.

Additionally, testing identified 287 prevalent SFGR cases, 626 probable SFGR cases, and 288 suspected SFGR cases and 239 prevalent ehrlichiosis cases, 384 probable ehrlichiosis cases, and 233 suspected ehrlichiosis cases. In total, 28.8% of individuals tested for SFGR and 35.2% of individuals tested for ehrlichiosis met the laboratory case definition of a suspected or probable case.

TPR was similar between seasonal periods for Lyme disease (4.1% during the high season and 4.8% during the low season), but it was greater during the high vs low season for SFGR (44.6% vs 39.2%; *P* = .01) and during the low vs high season for ehrlichiosis (37.9% vs 45.7%; *P* < .01). Compared with those without a positive test, patients testing positive for Lyme disease were younger with a median (IQR) age of 53 (30-62) years vs 50 (38-66) years (*P* = .04), whereas those testing positive for ehrlichiosis trended older with a median (IQR) age of 57 (41-69) years vs 53 (37-57) years (*P* = .03). Age was not significantly associated with SFGR positivity.

Of the 4577 individuals who were tested for SFGR, only 536 (11.8%) had paired acute and convalescent testing performed. A higher rate of paired testing was observed with ehrlichiosis (378 of 2507 [15.1%]) (Table 3). Individuals with paired testing tended to be older than those without paired testing for both SFGR (median [IQR] age, 57 [41-68] years vs 52 [35-66] years; *P* < .001) and ehrlichiosis (median [IQR] age, 56 [41-69] years vs 53 [37-67] years; *P* = .007). Additionally, individuals with an acute test result of greater than or equal to 1:64 were more than twice as likely to have a convalescent test ordered. Adherence to standard testing protocol was higher for Lyme disease, with only 295 individuals (2.9%) undergoing a Western blot without a preceding indeterminate or positive antibody test. Of these, 12 (4.1%) had positive IgM results and 1 (0.3%) had a positive IgG result (Table 4).

If individuals who only received an acute test experienced the same incidence rate as those with paired testing, we estimate that approximately 187 incident cases of SFGR and 151 incident cases of ehrlichiosis were not identified as a result of incomplete testing. Furthermore, because SFGR and ehrlichiosis have overlapping clinical disease spectra—and thus individuals presenting with typical symptoms (eg, fever, headache, myalgia) or suspected tick exposure should be tested for both diseases—we estimate that there were 2256 missing ehrlichiosis tests. Again, assuming similar incidence rates among individuals in whom SFGR was considered, but testing for ehrlichiosis was not performed, there would be an additional 158 missing incident cases, raising the estimate of total missed ehrlichiosis cases to 309.

## Discussion

Despite the increasing awareness of TBD as a growing public health threat, our study suggests that critical gaps remain in the routine surveillance systems underlying our knowledge of the spatial and clinical epidemiology of these diseases. The low rate of testing completeness, with only 1 in 10 individuals tested for SFGR having both acute and convalescent testing performed, severely limits the ability to distinguish incident infection from prior exposure. The paucity of confirmed diagnoses, both positive and negative, confounds prevention and control efforts, and introduces substantial misclassification bias into epidemiological and clinical research. Of particular concern, the apparent failure to consider ehrlichiosis as a potential cause of acute illness in approximately half of patients tested for SFGR raises serious doubts about our understanding of the burden of this disease. Improving the quality of routine surveillance data, through targeted education, systems-based interventions, and new testing modalities that do not require multiple visits, remains a key priority.

There are multiple potential explanations for the low level of testing completeness, including: (1) the resolution of clinical symptoms, either because of treatment or self-limited disease, which might reduce a patient’s motivation to return for a convalescent test, especially if incurring out-of-pocket costs; (2) the confirmation of alternative causes of illness over the interval between acute and convalescent testing; and (3) a general lack of frontline clinician knowledge regarding testing algorithms. One key to overcoming these issues is the development and more widespread adoption of acute stage diagnostics, such as PCR. We note that the US Food and Drug Administration recently approved a pan-*Rickettsia* real-time PCR assay and laboratory developed assays are available for *Ehrlichia*, but these tests are often inaccessible to frontline clinicians and even when available remain underused for reasons that merit further investigation.

While rates of testing completeness were relatively low—although still higher than similar data at the state or national level—our results identified notable trends in both testing and infection. First, the overall cohort in this study was relatively older and more female than previous national surveillance studies,^28,29^ although these characteristics are generally similar to the larger population seeking care at UNC Health. In the study sample, sex was not significantly associated with any disease positivity; however, we did observe that those positive for Lyme disease and ehrlichiosis were older than those testing negative. Whether this is because of differences in exposure risk, age-related clinical manifestations, or care-seeking behaviors is unclear. It is plausible that given the high levels of mild and even asymptomatic seroconversion previously seen in relatively young outdoor workers,^24,30^ we may be observing differential care-seeking patterns.

Another crucial finding is the high rate of testing for Lyme disease in contrast to the relative neglect of testing for ehrlichiosis, even among those tested for SFGR. The low rate of testing completeness and lack of consideration of ehrlichiosis likely resulted in a large number of unidentified cases, which would greatly impact surveillance data. While North Carolina reported a total of 1704 cases of SFGR and 372 cases of ehrlichiosis statewide during the study, our estimates suggest there might be an additional 187 SFGR and 309 ehrlichiosis cases from the UNC Health system alone. However, our estimates assume that individuals without paired testing experienced the same incidence rate as those with paired testing. Although, these groups differ significantly in ways that may affect the likelihood of a positive convalescent test and may ultimately overestimate the number of missed cases. In contrast, the high rate of Lyme disease testing may artificially inflate incidence rates in the area because of false-positive results, especially with the IgM assay, which represented most (69.7%) of the positive Western blot results.

Our study has several strengths including the large sample size abstracted from both outpatient and inpatient settings distributed throughout a relatively large geographic area. Furthermore, unlike previous analyses of notifiable disease reports, our study includes information about individuals who tested negative, which is information not normally available in these reports. Lastly, with high rates of SFGR and ehrlichiosis, and expanding Lyme in the western regions, North Carolina is a compelling setting to monitor trends in patterns of TBD testing.

### Limitations

This study had limitations. First, there was a lack of clinical data, such as the nature, onset, and duration of symptoms, which would have allowed us to more fully apply case definitions. Particularly, we were unable to identify cases in which initial testing was done within the first 7 days of illness onset, which is important because antibodies to many TBD may not yet be detectable. We were also unable to determine the frequency of alternative causes of febrile illness may have been present. For example, in some circumstances, individuals may have had a reactive SFGR titer, perhaps caused by prior exposure to *R. amblyommatis* and asymptomatic seroconversion, but ultimately have clinical disease caused by other bacterial or viral pathogens. Previous studies have demonstrated a high background rate among asymptomatic patients in the southeastern United States.^12^

Second, the absence of longitudinal data limited our ability to assess treatment decisions and, most importantly, clinical outcomes. Third, our study population may not be wholly representative of all patients seeking care for TBD in the area. Some patients may have been empirically treated without formal diagnostic testing based on the presence of typical symptoms and exposure history alone. Our sample may have been biased if individuals with standard disease presentation were more likely to have been empirically treated and less likely to be tested.

## Conclusions

These findings suggest that most patients suspected of having TBD did not have testing performed in accordance with established guidelines, which substantially limits our understanding of TBD epidemiology. Furthermore, there appears to be a large discrepancy between the local burden of disease and the testing that is performed. These findings underscore the need for better diagnostics and active surveillance programs, particularly for SFGR and ehrlichiosis, to more accurately identify the spatial distribution and risk factors for infection.

## Notes

Supplement. **eTable 1.** Annual Trends in Diagnostic Testing and Test Positivity for Tick-Borne Disease **eTable 2.** Total Number of Individuals Tested for Lyme Disease and Testing Results
