# Errors in Title and Byline

**DOI:** 10.1001/jamanetworkopen.2022.20227

**Published:** 2022-06-13

In the Original Investigation titled “Patterns of Testing for Tick-Borne Diseases and Implications for Surveillance in the Southeastern US,”^1^ published May 16, 2022, the word “of” was missing in the title and degrees were missing for 7 authors. The author degrees have been updated to Brandon D. Hollingsworth, PhD; Haley Abernathy, BS; Aidin Alejo, BS; Victor Arahirwa, BS; Odai Mansour, BS; John Schmitz, PhD; and Carl Williams, DVM. This article has been corrected.^1^
