# Supplementary material for: Patterns Testing for Tick-Borne Diseases and Implications for Surveillance in the Southeastern US
Source: JAMA Netw Open. 2022 May 16;5(5):e2212334. doi: 10.1001/jamanetworkopen.2022.12334 (PMC9112065; doi:10.1001/jamanetworkopen.2022.12334)
Supplement: Supplement. — eTable 1. Annual Trends in Diagnostic Testing and Test Positivity for Tick-Borne Disease eTable 2. Total Number of Individuals Tested for Lyme Disease and Testing Results [file jamanetwopen-e2212334-s001.pdf]

Supplemental Online Content

Brown Marusiak A, Hollingsworth BD, Abernathy H, et al. Patterns testing for tick-borne diseases and implications for surveillance in the southeastern US. *JAMA Netw Open*. 2022;5(5):e2212334. doi:10.1001/jamanetworkopen.2022.12334

eTable 1. Annual Trends in Diagnostic Testing and Test Positivity for Tick-Borne Disease

eTable 2. Total Number of Individuals Tested for Lyme Disease and Testing Results

This supplemental material has been provided by the authors to give readers additional information about their work.

© 2022 Brown Marusiak A et al. *JAMA Network Open.*

eTable 1. Annual Trends in Diagnostic Testing and Test Positivity for Tick-Borne Disease

	Lyme		SFGR			Ehrlichiosis		
	Total	TPR (%)	Total	TPR (%)	Incident Cases	Total	TPR (%)	Incident Cases
**2017**	3727	4.5	1621	32.9	5	724	16.7	3
**2018**	3576	3.0	1939	31.9	5	1590	36.1	10
**2019**	3415	4.5	1471	54.0	13	1100	47.6	7
**2020**	2691	4.1	1135	63.9	24	978	53.2	7

Abbreviations: SFGR = Spotted Fever Group Rickettsiosis, TPR = test positivity rate

© 2022 Brown Marusiak A et al. *JAMA Network Open.*

eTable 2. Total Number of Individuals Tested for Lyme Disease and Testing Results

	Antibody		IgG	IgM	PCR	Overall
Individuals	9858		614	614	222	10208
Total Tests	10460		666	666	224	12016
Positive Tests	197 (2.0)		26 (4.2)	88 (14.3)	0 (0.0)	311 (3.1)
Negative Tests	10240		639	557	224	11680
Equivocal	23		1	1	0	25
Confirmed Cases	---		---	---	---	76 (0.8)

Abbreviations: IgG = immunoglobulin G, IgM = immunoglobulin M, PCR = polymerase chain reaction

© 2022 Brown Marusiak A et al. *JAMA Network Open.*
